# Molecular identification and *in vitro* evaluation of probiotic functional properties of some Egyptian lactic acid bacteria and yeasts

**DOI:** 10.1186/s43141-021-00212-4

**Published:** 2021-08-05

**Authors:** Al-Shimaa Ibrahim Ahmed, Gihan Mohamed El Moghazy, Tarek Ragab Elsayed, Hanan Abdel Latif Goda, Galal Mahmoud Khalafalla

**Affiliations:** 1 Agricultural Research Centre, Regional Centre for Food and Feed, Giza, 12619 Egypt; 2 Agricultural Microbiology Department, Faculty of Agriculture, Cairo University, Giza, 12613 Egypt

**Keywords:** *Enterococcus mediterraneensis*, *Lactobacillus fermentum*, *Streptococcus lutetiensis*, Probiotics, Functional characteristics

## Abstract

**Background:**

The health-promoting effects along with global economic importance of consuming food products supplemented with probiotic microorganisms encouraged the researchers to discover new probiotics.

**Results:**

Fourteen lactic acid bacterial isolates were identified as *Enterococcus mediterraneensis, Lactobacillus fermentum,* and *Streptococcus lutetiensis* by 16S rRNA gene sequencing, and *in vitro* characterized for their actual probiotic potential. All *E. mediterraneensis* isolates were resistant to clindamycin, whereas *Lb. fermentum* isolates were resistant to ampicillin, clindamycin, and vancomycin. The *E. mediterraneensis* and *Lb. fermentum* isolates displayed high overall digestive survival, ranged from 1.35 ± 0.06 to 32.73 ± 0.84% and from 2.01 ± 0.01 to 23.9 ± 1.85%, respectively. All isolates displayed cell surface hydrophobicity, ranged between 15.44 ± 6.72 and 39.79 ± 2.87%. The strongest auto-aggregation capability, higher than 40%, was observed for most *E. mediterraneensis* and *Lb. fermentum* isolates. The *E. mediterraneensis* isolates (L2, L12, and L15), *Lb. fermentum* (L8, L9, and L10), and *Strep*. *lutetiensis* (L14) exhibited the greatest co-aggregation with *Salmonella typhimurium*, *Escherichia coli* O157:H7, *Staphylococcus aureus,* and *Bacillus cereus*. Fifty-seven and fourteen hundredth percent of *E. mediterraneensis* isolates could be considered bacteriocinogenic against *E. coli* O157:H7, *B. cereus,* and *S. aureus*.

**Conclusion:**

This study is the first one to isolate *Enterococcus mediterraneensis* in Egypt and to characterize it as new species of probiotics globally. According to the results, *E*. *mediterraneensis* (L2, L12, and L15), *Lb. fermentum* (L8, L9, and L10), and *Strep. lutetiensis* (L14) are the most promising *in vitro* probiotic candidates.

## Background

Increasing the consumers’ consciousness of health-promoting effects of functional foods encouraged both researchers and manufacturers to enhance the production of these food types. The Academy of Nutrition and Dietetics defines functional foods as “whole foods along with fortified, enriched, or enhanced foods that have a potentially beneficial effect on health when consumed as part of a varied diet on regular basis at effective levels based on significant standards of evidence” [1]. According to this definition, the functional foods could be supplemented with active substances of beneficial biological activity. The food formulations supplemented with probiotics is considered as an important research area for the development of functional food production and marketing. Globally, an increase from 3.3 to 7 billion dollars was expected for the market of probiotic dietary supplements throughout the period from 2015 to 2025 [2].

In 2001, the joint Food and Agriculture Organization of the United Nations (FAO) and World Health Organization (WHO) Working Group defined probiotics as “live microorganisms which when administered in adequate amounts confer a health benefit on the host” [3]. The main microbial genera used as probiotics include *Lactobacillus, Lactococcus, Enterococcus, Pediococcus, Propionibacterium, Bifidobacterium, Escherichia, Bacillus, Staphylococcus,* and some yeast genera, mainly *Saccharomyces* [4]*.*

For at least the last 15 years, the awareness to use probiotics as an environmentally friendly alternative in food and feed production to improve human and animal health is growing. Several *in vitro* studies confirmed the health-promoting effects of probiotics comprising antiobesity, anticancer activity, antioxidant activity, inflammatory intestine disease mitigation, food allergy alleviation, improving lactose tolerance, suppression of microbial pathogens, strengthened innate immunity, and serum cholesterol lowering [5, 6]. Also, probiotic microorganisms are used as an alternative to antibiotics to reduce the drug resistance resulting from mis and overuse of antibiotics for disease treatment in humans and animals [7]. Furthermore, improper drug withdrawal times and addition of unsuitable doses of antibiotics to animal feed for growth promotion led to presence of antibiotic residues in meat and milk. When humans consume these foods, the antibiotic residues will accumulate in the body and may cause numerous side effects as transfer of antibiotic-resistant bacteria to humans, allergy, immunopathological effects, kidney failure, hepatotoxicity, bone marrow toxicity, and reproductive disorders [8]. Generally, the beneficial effects of probiotics are depending on the microbial type, their delivery method, host response, and interaction with other microbes [9].

The probiotic microorganisms should meet the quality assurance criteria for probiotic selection including non-pathogenic and toxigenic activity, gastrointestinal juice tolerance, adherence ability to intestinal epithelial cells, auto- and co-aggregation activities, and antibiotic resistance [10, 11]. In addition to the above, on the commercial scale, the probiotics must retain their vitality and stability during food processing and storage.

From the aforementioned, it is evident the importance of probiotics healthily and economically, which had a great impact for the existence of continuous attempts to discover new probiotics depending on strain selection criteria and their survival during biomass production and storage. Therefore, the main objective of this study was evaluating the potential of some Egyptian lactic acid bacteria and yeast isolates to be probiotic candidates through characterization of their safety and functional properties.

## Methods

### Sample collection

Different samples including animal feed ingredients, dairy products, and meat products were congregated from local markets in Giza Governorate, Egypt to isolate probiotic lactic acid bacteria (LAB) and yeasts. The collected twenty samples of common animal feed ingredients included fish meal (3 samples), corn gluten meal (5 samples), soybean meal (7 samples), and sunflower meal (5 samples). Also, two samples from kareish cheese (local type of fresh soft cheese manufactured from skimmed cow milk), two samples from rayeb milk, one sample from yoghurt, and two samples from processed meat (frankfurter) were screened for the presence of probiotic LAB and yeasts.

### Determination and isolation of potential probiotic LAB and yeasts

The pour plate method was applied to determine and isolate the potential probiotics. de Man, Rogosa, and Sharpe (MRS) agar (Lab M, Neogen Company, UK) at 37 °C/48 h and rose bengal agar (Lab M, Neogen Company, UK**)** at 30 °C/48 h were used to determine total LAB and yeasts, respectively.

The potential probiotic LAB and yeasts were isolated using modified MRS agar and rose bengal agar. This modification was represented by lowering pH to around 2.5 and addition of bile salt with a concentration of 0.3%. The bacterial colonies from modified MRS agar plates were selected randomly for morphological and biochemical characterization. Only pure Gram-positive and catalase-negative isolates were considered as potential probiotic LAB. Other bacterial isolates, with different profile according to Gram staining and catalase test, were considered as not LAB. All bacterial and yeast isolates were preserved at − 20 °C using MRS broth and rose bengal broth supplemented with 20% glycerol.

### Molecular identification of isolated bacteria and yeasts

All bacterial and yeast isolates were identified according to 16S rRNA and ITS gene sequencing, respectively. All lactic acid bacterial isolates were grown in MRS broth for 24 h at 37 °C. The other bacterial isolates were cultivated in tryptone glucose yeast extract broth **(**Lab M, Neogen Company, UK**)** at 37 °C/24 h. The yeast isolates were grown in rose bengal broth for 24 h at 30 °C. The cells were harvested by centrifugation at 12,000 g for 5 min. After washing the pellets for three times using 0.85% NaCl solution, genomic DNA was extracted using GeneJET Genomic DNA purification Kit (ThermoFisher Scientific, Republic of Lithuania). DNA yields and purity were checked using both UV-Vis NanoDrop spectrophotometer (NanoDrop 2000, ThermoFisher Scientific, Germany) and agarose gel electrophoresis (Bio-rad, USA).

To evaluate the genotypic diversity, the BOX-PCR fingerprints of bacteria were generated for all bacterial isolates using BOXA1R primer (CTACGGCAAGGCGACGCTGACG) [12]. The PCR conditions were initial denaturation step at 95 °C for 12 min, followed by 30 cycles of 95 °C for 30 s, 55 °C for 1 min and 72 °C for 1 min, and one extension step at 72 °C for 10 min at the end of PCR reaction. The PCR tubes were kept at − 20 °C until used. Eight microliters of the PCR product were separated by 1.5% agarose gel electrophoresis in 0.5 X TBE-buffer for 4 h (50 V). The BOX-PCR fingerprints patterns were checked and compared visually.

The 16S rRNA gene fragments of 9 LAB isolates were amplified using the universal primers F-27 (5′-AGAGTTTGATCMTGGCTCAG-3′) and R-1494 (5′-CTACGGYTACCTTGTTACGAC-3′). For yeast isolates, the internal transcribed spacer (ITS) region was amplified using the universal primers ITS1 (5′-CTTGGTCATTTAGAGGAAGTAA-3′) and ITS4 (5′-TCCTCCGCTTATTGATATGC-3′). The amplification step was performed using Thermal cycler PCR (Bio-rad T100, USA). The PCR conditions for 16S rRNA gene were initial denaturation step at 95 °C for 12 min, followed by 30 cycles of 94 °C for 1 min, 56 °C for 1 min and 72 °C for 2 min, and one extension step at 72 °C for 10 min at the end of PCR reaction. The PCR conditions for the ITS gene were initial denaturation step at 95 °C for 12 min, followed by 30 cycles of 95 °C for 30 s, 55 °C for 30 s and 72 °C for 1 min, and one extension step at 72 °C for 10 min at the end of PCR reaction. The PCR products were checked via agarose gel electrophoresis then purified using gel extraction kit and sequenced by Macrogen, Inc., Seoul, South Korea using automatic ABI 370 × 1 DNA Sequencer (Applied Biosystem, USA). The sequences were analyzed applying BLAST V2.0 software (http://www.ncbi.nlm.nih.gov/BLAST/).

### Phylogenetic analysis of bacterial and yeast isolates

The evolutionary history was inferred using the neighbor-joining method. The tree was computed using the maximum composite likelihood method. Two phylogenetic trees were constructed. The first one involved 30 bacterial nucleotide sequences of which 9 sequences of 16S rRNA gene amplified from bacterial isolates, while 21 sequences representing the most similar hits were obtained from the NCBI GeneBank database. The second tree involved 9 yeast sequences of which 4 sequences of ITS regions amplified from yeast isolates, while 5 sequences were from the NCBI GeneBank database. Evolutionary analyses were conducted using MEGA5 software.

### Characterization of safety properties

#### Characterization of pathogenicity

The pathogenicity of isolated probiotics was evaluated through testing the ability of isolates for blood hemolysis. The inoculum from freshly prepared slant culture of LAB and yeasts was streaked on the blood agar surface with incubation at 37 °C for LAB or 30 °C for yeasts/24–48 h. After the incubation, the cultures were tested for blood hemolysis pattern (α, β, or γ hemolysis). According to the results, all yeast isolates were excluded from this study.

#### Characterization of antibiotic susceptibility

The antibiotic resistance of isolated probiotic LAB was examined through determination of minimum inhibitory concentration (MIC) of 9 antibiotics from different antibiotic classes with different antibacterial mode of action (Table 1).

**Table 1:** Selected antibiotics to evaluate the antibiotic susceptibility of isolated LAB

Antibiotic class	Antibiotic	Mode of action	Diluent for stock solution
Beta-lactams	Penicillins: ampicillin (AM)	Inhibition of cell wall synthesis	Phosphate buffer
Beta-lactams	Cephalosporins: 5th generation: ceftolozane (CEF)	Inhibition of cell wall synthesis	Water
Macrolides	Clindamycin (CLI)	Inhibition of protein synthesis	Water
Tetracycline	Tetracycline (TE)	Inhibition of protein synthesis	Water
Quinolones	4th generation: moxifloxacin (MXF)	Inhibition of DNA gyrase	Water
Aminoglycosides	Neomycin (NEO)	Inhibition of protein synthesis	Water
Sulphonamides	Sulphamethoxazole (SXT)	Inhibition of folic acid metabolism	Water
Glycopeptides	Vancomycin (VAN)	Inhibition of cell wall synthesis	Water
Synthetic antibiotics	Chloramphenicol (CHL)	Inhibition of protein synthesis	Ethanol 95%

The MIC of selected antibiotics was determined applying the broth dilution method. Each antibiotic was two-fold serially diluted, from 128 to 1 μg/mL, in MRS broth. The isolated LAB were grown at 37 °C for 24 h in MRS broth supplemented with the given concentrations of antibiotic. After incubation, the calculated MIC was compared with antibiotic breakpoint specified by the European Food Safety Authority (EFSA, 2012) [13] or Clinical and Laboratory Standards Institute (CLSI, 2014 and 2017) [14, 15] to categorize the isolates as susceptible or resistant to the tested antibiotic.

### Characterization of functional properties

Gamma hemolytic (nonhemolytic) isolates were assessed for their functional properties. For all characterization tests, the inoculum from 24 h slant culture of LAB was inoculated in modified MRS broth. The broth cultures grown for 24 h were centrifuged at 1270 g for 10 min to collect the microbial cells, which were washed twice with sterile phosphate buffer solution (PBS) (pH 7). The probiotic reference strains *Lactobacillus acidophilus* ATCC 20552 was used for comparative probiotic characterization. All tests were conducted in three replicates.

#### Phenol tolerance

Ability of isolated LAB to survive in the presence of phenol was examined. The bacterial cultures with a count of 10^7^–10^8^ cfu/mL were inoculated in MRS broth containing 0.4% phenol, and incubated at 37 °C for 24 h. At 0 time and after 24 h of incubation, the bacterial count was determined employing the pour plate method [16]. The survival rate was calculated according to the formula:

Survival rate (%) = (log cfu *N*_*t*_ / log cfu *N*_0_) × 100

As *N*_*t*_ and *N*_0_ represent the surviving count after 24 h and initial count, respectively.

#### Survival in a simulated human digestive system

Influence of human digestion process on the viability of potential probiotic isolates was assessed applying the *in vitro* gastric and intestinal digestion successively [17]. The gastric digestion was performed using the simulated gastric juice prepared by dissolving 0.13 g NaCl, 0.024 g KCl, 0.64 g NaHCO_3,_ and 0.3 g bile salts (Oxford Laboratory, India) in 50 mL of sterile distilled water. After adjusting the pH to 2.5, 0.1 g pepsin (pepsin 1: 3000, Oxford laboratory, India) was dissolved, and the total volume was completed to 100 mL [18]. The collected cells were resuspended in simulated gastric juice and incubated at 37 °C/2 h using an orbital shaker at 200 rpm. The initial count (*C*_0_) and the surviving count after the gastric digestion were determined by the pour plate method using MRS agar.

The harvested cells from gastric juice were washed in PBS and resuspended in the same volume of simulated intestinal juice formulated by dissolving 0.68 g monobasic potassium phosphate (KH_2_PO_4_) in 25 mL sterile distilled water. After adjusting the pH to 6.8, 1 g pancreatin (Ambezim-G, Global Napi Pharmaceuticals, Egypt. Each film-coated tablet contains 5 mg trypsin and 5 mg chymotrypsin which is equivalent to 200,000 proteolytic units) was dissolved, and the total volume was completed to 100 mL [19]. After incubation at 37 °C/4 h using an orbital shaker at 200 rpm, the count after the intestinal step (*C*_*i*_) was determined as mentioned before. Overall digestion survival (ODS) was calculated and expressed in percentage according to the formula: ODS% = (*C*_*i*_ / *C*_0_) × 100

#### Cell surface hydrophobicity assay

Cell surface hydrophobicity of the isolates was assessed by measuring the microbial adhesion to hydrocarbons [16]. The method was based on the cell affinity to xylene in a two-phase system. The harvested cells were resuspended in PBS (pH 7) to an OD_600_ of 0.6 ± 0.02 using the UV/Vis spectrophotometer (Metash UV-800, Shanghai, China) to standardize the count to approximately 10^7^ to 10^8^ cfu/mL. Two milliliters of the microbial suspension were mixed with 2 mL of xylene for 2 min using the vortex mixer. After 30 min in steady state to allow the separation of two phases, OD_600_ of the aqueous phase was measured. The percentage of cell surface hydrophobicity (CSH) was calculated according to the following equation:

CSH% = [(*A*_0_ − *A*)/*A*_0_] × 100

where *A*_0_ is the OD_600_ before mixing, and *A* is the OD_600_ after 30 min of mixing.

#### Auto-aggregation assay

Ability of cells for self-binding (auto-aggregation) was evaluated by resuspending the obtained pellet in PBS (pH 7), followed by adjusting the OD_600_ at 0.6 ± 0.02. During incubation at 37 °C and without shaking the cell suspension, OD_600_ was measured at time intervals of 1, 2, 3, and 24 h in the upper layer [16]. The percentage of auto-aggregation was calculated applying the following equation: Auto-aggregation% = [1 − (*A*_*t*_/*A*_0_)] × 100

as *A*_0_ and *A*_*t*_ represent absorbance (OD_600nm_) at 0 time and after selected time of incubation, respectively.

#### Co-aggregation assay

The ability of LAB isolates to aggregate with the bacterial pathogens was tested. The selected pathogens comprised *Salmonella enterica* subsp. *enterica* serovar Typhimurium ATCC 14028 (*Sal. typhimurium*), *Escherichia coli* O157:H7 BCR 594 (*E. coli*), *Staphylococcus aureus* subsp. *aureus* ATCC 25923 (*S*. *aureus*) and *Bacillus cereus* BCR 528 (*B. cereus*). Two milliliters of each lactic acid bacterial suspension prepared in PBS were mixed for 10 s by a vortex mixer with equal volume of the various suspensions of pathogenic bacteria (10^7^–10^8^ cfu/mL). During incubation of each mixture at 37 °C/24 h, the OD_600nm_ was measured at time intervals of 2, 4, and 24 h [16]. The co-aggregation ability was determined as follows:

Co-aggregation% = [(*A*_mix0_ – *A*_mixt_) / *A*_mix0_] × 100

as *A*_mix0_ and *A*_mix*t*_ represent absorbance (OD_600nm_) at 0 time and after selected time of incubation, respectively.

#### Antimicrobial activity assay

All previous bacterial pathogens were used as indicator microorganisms to evaluate the antibacterial activity of isolated probiotic candidates. To assess the antagonistic activity of isolated LAB, the broth cultures were prepared under both aerobic and anaerobic conditions. After cultivation for 24 h at 37 °C under both conditions, the cells were removed from the cultures by centrifugation at 1270 g for 15 min, followed by filtration of the supernatant through a cellulose acetate membrane filter with a pore size of 0.22 μm. The filtrate from broth cultures prepared aerobically was considered as crude extract that could contain H_2_O_2_, organic acids, and bacteriocins (Extract 1). On the other hand, the filtrate from broth cultures prepared anaerobically was considered as crude extract that could contain organic acids and bacteriocins only (Extract 2). To eliminate the inhibitory effect of organic acids in Extract 2, the cell-free supernatant was neutralized with 1 M NaOH. The resulting is referred to as a crude bacteriocin, if produced (Extract 3). The antibacterial activity of all extracts was screened applying the well–diffusion assay (cup–plate method) [20], in which 200 μL of the extract was added in a well of 10 mm diameter made into the plate containing agar medium inoculated with the test microorganism. The plates were incubated at 37 °C for 24 h. Growth inhibition appeared as a measurable clear zone around the disc.

### Statistical analysis

Data were expressed as mean ± standard deviation (SD). The differences between the isolates were statistically evaluated by one-way analysis of variance (ANOVA) followed by Tukey’s multiple comparison test using Graph Pad Prism program 7.0. *P* < 0.05 was considered significant.

## Results

### Determination and isolation of potential probiotic LAB and yeasts

Neither LAB nor yeasts were isolated from all collected animal feed ingredients. On the other hand, LAB, yeasts and their corresponding acid–bile salt-tolerant cells were determined and isolated from tested food samples.

The results revealed infrequent presence of acid–bile salt-tolerant bacteria in food samples. The highest percentage was recorded in yoghurt (3.0%), followed by rayeb milk (0.02–0.03%). The lowest incidence was in kareish cheese and frankfurter with a percentage of 2.0 × 10^-6^ to 4.0 × 10^-5^% and 3.0 × 10^-5^ to 3.0 × 10^-4^%, respectively.

The acid–bile salt-tolerant yeasts were determined only in kareish cheese in the range of 3.0 × 10^-5^ to 2.0 × 10^-4^%.

A total of 16 and 4 isolates, characterized as acid and bile salt-tolerant bacteria (designated as L1–L16) and yeasts (designated as Y1–Y4), respectively, were selected randomly. The bacterial isolates included 2, 4, and 9 isolates from kareish cheese, rayeb milk, and frankfurter samples, correspondingly. Also, 1 isolate was obtained from the yoghurt sample. Gram staining and catalase test categorized the isolates into 3 classes: (i) G^+^ and catalase negative cocci (9 isolates), (ii) G^+^ and catalase negative rods (5 isolates), and (iii) G^+^ and catalase positive sporulated rods (2 isolates).

Morphologically, the 4 yeast isolates, obtained from kareish cheese, were oval-shaped cells.

### Bacterial fingerprints and genotypic diversity

**Fig. 1:**
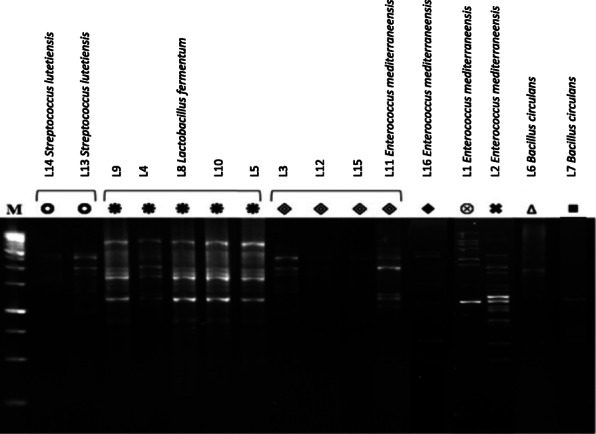
BOX-PCR fingerprints of 16 bacterial isolates. Fingerprint profiles labeled with the same symbol are identical. M, 1 Kb ladder

BOX-PCR fingerprints were generated for all acid–bile salt-tolerant bacterial isolates (16 isolates) to show their genotypic diversity. Three major genotypes with multiple isolates were detected in addition to 5 unique fingerprint profiles each with only one single pattern (Fig. 1). Identical fingerprint profiles were recorded among the isolates (L13 and L14) and between the isolates (L4, L5, L8, L9, and L10) and (L3, L11, L12, and L15). At least one representative isolate from each fingerprint profile was identified based on the sequence of 16S rRNA gene.

### 16S rRNA-based identification and phylogenetic analysis of bacterial isolates

**Fig. 2:**
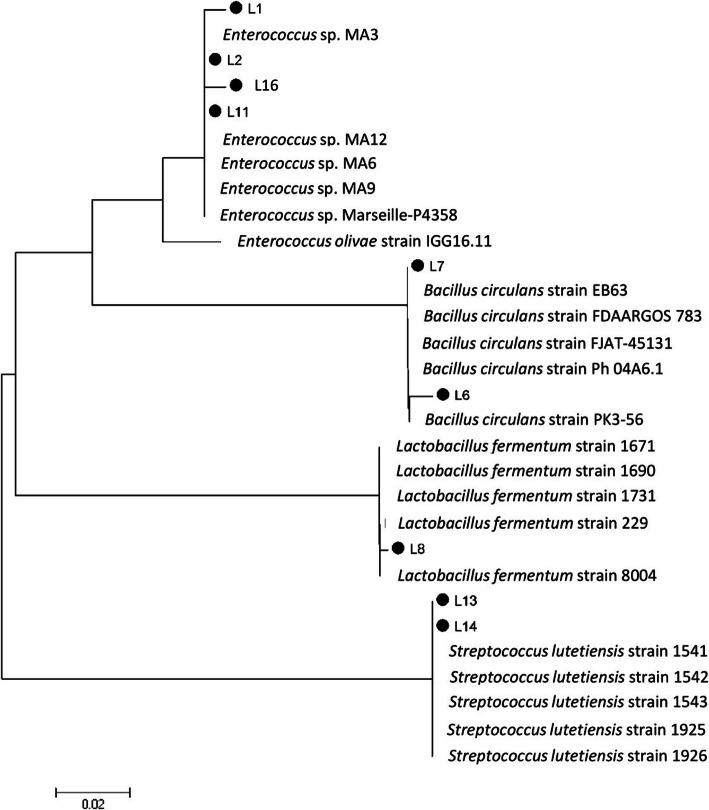
A neighbor-joining phylogenetic tree based on 16S rRNA gene sequences of 9 LAB isolates (dark circles) with the closest hits obtained from the NCBI GeneBank

Nine bacterial isolates, representing different BOX-PCR fingerprint profiles, were identified based on the sequencing of 16S rRNA gene. The phylogenetic analysis with closest hits obtained from the NCBI GeneBank is presented in Fig. 2. The 16S rRNA sequence revealed that the isolates L1, L2, L16, and L11 showed 99% similarity to *Enterococcus mediterraneensis* (*E*. *mediterraneensis*), the isolates L6 and L7 were 99 and 100% similar to *Bacillus circulans* (*B. circulans*) respectively, while L8 showed 99% similarity to *Lactobacillus fermentum* (*Lb*. *fermentum*). The isolates L13 and L14 showed 99% similarity to *Streptococcus lutetiensis* (*Strep. lutetiensis*).

### ITS-based identification and phylogenetic analysis of yeast isolates

**Fig. 3:**
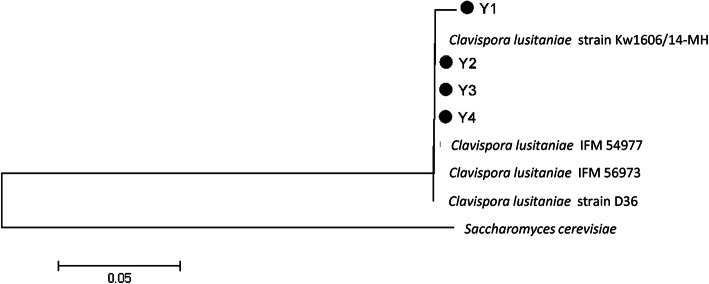
A neighbor-joining phylogenetic tree based on ITS gene sequences of 4 yeast isolates (dark circles) with the closest hits obtained from the NCBI GeneBank

The phylogenetic analysis of yeast isolates based on the sequencing of the ITS region is presented in Fig. 3. All yeast isolates were identified as *Clavispora lusitaniae* with similarity ranged from 98.6 to 99.7% (Table 2).

**Table 2:** Accession numbers and closest hits of LAB and yeast isolates

Isolate no.	Source of isolation	Closest hit	Gene identity (%)	GenBank accession number
**L1**	Rayeb milk	*Enterococcus mediterraneensis* MA3	**99**	MT860218
**L2**	Rayeb milk	*Enterococcus mediterraneensis* MA12	**99**	MT860219
**L16**	Kareish cheese	*Enterococcus mediterraneensis* MA12	**99**	MT860213
**L11**	Frankfurter	*Enterococcus mediterraneensis* MA12	**99**	MT860215
**L6**	Frankfurter	*Bacillus circulans* strain PK3-56	**99**	MT860220
**L7**	Frankfurter	*Bacillus circulans* strain EB63	**100**	MT860221
**L8**	Frankfurter	*Lactobacillus fermentum* strain 8004	**99**	MT860214
**L13**	Frankfurter	*Streptococcus lutetiensis* strain 1541	**99**	MT860216
**L14**	Frankfurter	*Streptococcus lutetiensis* strain 1541	**99**	MT860217
**Y1**	Kareish cheese	*Clavispora lusitaniae* IFM 549787	**98.6**	MT880128
**Y2**	Kareish cheese	*Clavispora lusitaniae* IFM 56973	**99.7**	MT880130
**Y3**	Kareish cheese	*Clavispora lusitaniae* IFM 56973	**99.45**	MT880132
**Y4**	Kareish cheese	*Clavispora lusitaniae* IFM 56973	**99.17**	MT880131

The sequences of LAB and yeast isolates were deposited on the GeneBank under accession numbers presented in Table 2.

### Characterization of safety properties

#### Characterization of pathogenicity

The blood hemolysis test was conducted to characterize the pathogenicity of catalase-negative bacteria (14 isolates) and all yeast isolates. The results confirmed that the colonies of all tested bacterial isolates were not surrounded by clear or greenish zone on blood agar indicating that these isolates are gamma hemolytic or non-hemolytic bacteria. Conversely, the 4 yeast isolates were characterized as alpha hemolytics.

The gamma hemolytic lactic acid bacteria were tested to define their antibiotic susceptibility profile and characterized functionally *in vitro* to evaluate their actual probiotic potential.

#### Characterization of antibiotic susceptibility

The antibiotic susceptibility of all 14 LAB isolates was assessed through determination of minimum inhibitory concentration (MIC) of 9 antibiotics (Table 3). According to the antibiotic breakpoints reported by EFSA (2012) and CLSI (2014 and 2017), the antibiotic resistance of putative probiotic LAB was specified. All *E. mediterraneensis* isolates were sensitive to neomycin and vancomycin and resistant to clindamycin. Alternatively, 14.29% were resistant to ampicillin and ceftolozane, 42.86% to tetracycline and chloramphenicol, and 57.14% to moxifloxacin and sulphamethoxazole. All *Lb. fermentum* isolates were sensitive to ceftolozane, neomycin, and sulphamethoxazole; and resistant to ampicillin, clindamycin, and vancomycin. On the other hand, 80% were resistant to chloramphenicol and 40% to tetracycline and moxifloxacin. The two isolates of *Strep. lutetiensis* (L13 and L14) were resistant to ampicillin, clindamycin, tetracycline, moxifloxacin, sulphamethoxazole, and vancomycin, and completely susceptible to ceftolozane, neomycin, and chloramphenicol.

**Table 3:** Minimum inhibitory concentration (μg/mL) of 9 antibiotics against isolated LAB

Isolate no.	Isolate identification	MIC (μg/mL)								
		AM^**2**^	CEF^**3**^	CLI^**4**^	TE^**5**^	MXF^**6**^	NEO^**7**^	SXT^**8**^	VAN^**9**^	CHL^**10**^
PRS^1^	*Lb. acidophilus* ATCC 20552	16R^11^	16S^12^	2R	2S	4R	32R	4R	2R	64R
L1	*E. mediterraneensis*	4 S	2 S	8 R	4 S	2I^13^	2S	2 S	2 S	4 S
L2		2 S	2S	2R	4S	2I	2S	4R	4S	8S
L3		2 S	2S	2R	2 S	2I	2S	4R	2S	128R
L11		8 S	64R	2R	64R	8R	8S	4R	2S	32R
L12		2 S	2S	2R	32R	8R	2S	2S	2S	16I
L15		8 S	8S	2R	8I	4R	8S	2S	4S	2S
L16		32R	32S	2R	32R	4R	4S	4R	2S	32R
L4	*Lb. fermentum*	2R	4S	2R	2S	2S	8S	2S	8R	2S
L5		2R	2S	2R	4S	2S	2S	2S	8R	4R
L8		4R	2S	2R	8R	4R	2S	2S	2R	4R
L9		8R	2S	2R	2S	2S	8S	2S	2R	8R
L10		8R	32S	2R	32R	8R	8S	2S	8R	32R
L13	*Strep. lutetiensis*	4R	2S	4R	16R	8R	32S	16R	8R	2S
L14		4R	2S	4R	16R	8R	32S	16R	8R	2S

1: probiotic reference strain. 2: ampicillin. 3: ceftolozane. 4: clindamycin. 5: tetracycline. 6: moxifloxacin. 7: neomycin. 8: sulphamethoxazole. 9: vancomycin. 10: chloramphenicol. 11: resistant. 12: sensitive. 13: intermediate resistant

### Characterization of functional properties

#### Phenol tolerance

In the presence of 0.4% phenol, the survival rate of *E. mediterraneensis* and *Lb. fermentum* isolates ranged from 64.9 ± 1.41 to 102.97 ± 0.67% and from 52.59 ± 1.26 to 99.34 ± 0.93%, correspondingly (Table 4). The two isolates of *Strep. lutetiensis* had phenol tolerance higher than 80%.

**Table 4:** Phenol tolerance of isolated LAB

Isolate no.	Isolate identification	Survival rate (%)	Isolate no.	Isolate identification	Survival rate (%)
PRS^1^	*Lb. acidophilus* ATCC 20552	99.25 ± 0.88	L4	*Lb. fermentum*	78.65 ± 1.48
L1	*E. mediterraneensis*	99.52 ± 0.67*	L5		52.59 ± 1.26
L2		99.45 ± 0.77*	L8		85.46 ± 1.14*
L3		64.9 ± 1.41	L9		99.34 ± 0.93*
L11		89.51 ± 1.54*	L10		84.77 ± 0.13*
L12		87.79 ± 0.77*	L13	*Strep. lutetiensis*	80.34 ± 1.59
L15		87.67 ± 1.14*	L14		98.83 ± 0.37*
L16		102.97 ± 0.67*			

From the total isolates, 28.57% had the ability to grow significantly better than the reference strain with a survival rate ranging from 99.34±0.93 to 102.97±0.67%. These isolates were *E. mediterraneensis* (L1, L2, and L16), and *Lb. fermentum* (L9). The isolates L3, L4, and L5 showed the least tolerance to phenol with a survival rate ranged from 52.59±1.26 to 78.65±1.48%.

It was observed that the phenol, with a concentration of 0.4%, has rather stimulating effect on the growth of one isolate of *E. mediterraneensis* (L16 isolated from kareish cheese) as the survival rate reached more than 100%.

#### Survival in a simulated human digestive system

The results in Table 5 elucidated that 57.14% of *E. mediterraneensis* isolates displayed ODS ranged between 11.43 ± 1.29 and 32.73 ± 0.84%, whereas 80% of *Lb*. *fermentum* isolates had ODS in a range from 11.38 ± 0.33 to 23.9 ± 1.85%.

**Table 5:** Overall digestion survival (ODS) of potential probiotic LAB

Isolate no.	Isolate identification	ODS (%)	Isolate no.	Isolate identification	ODS (%)
PRS^1^	*Lb. acidophilus* ATCC 20552	4.68 ± 0.74	L4	*Lb. fermentum*	11.99 ± 1.33*
L1	*E. mediterraneensis*	2.09 ± 0.3	L5		14.77 ± 0.32*
L2		32.73 ± 0.84*	L8		23.9 ± 1.85*
L3		11.43 ± 1.29*	L9		2.01 ± 0.01
L11		15.39 ± 1.56*	L10		11.38 ± 0.33*
L12		13.35 ± 0.35*	L13	*Strep. lutetiensis*	11.02 ± 0.43*
L15		1.56 ± 0.16	L14		9.42 ± 0.13
L16		1.35 ± 0.06			

1: probiotic reference strain. Data are expressed as mean ± SD (standard deviation). * *P* values were ˂ 0.05.

It was revealed that the superior survival rates of 32.73 ± 0.84 and 23.9 ± 1.85%, through gastric and intestinal transit, appeared with *E. mediterraneensis* (L2) and *Lb*. *fermentum* (L8), respectively. The isolates of *E. mediterraneensis* (L3, L11, and L12), *Lb*. *fermentum* (L4, L5, and L10), and *Strep. lutetiensis* (L13) displayed lower survival significantly.

#### Cell surface hydrophobicity assay

The results shown in Table 6 indicated that all LAB isolates revealed a different degree of hydrophobicity in xylene which ranged between 15.44 ± 6.72 and 39.79 ± 2.87%. The isolates of *E. mediterraneensis* (L3), *Lb*. *fermentum* (L5), and *Strep*. *lutetiensis* (L13) had strong hydrophobicity represented by 37.87 ± 9.71%, 38.88 ± 8.07%, and 39.79 ± 2.87%, correspondingly comparing to the reference strain (26.44 ± 2.31%).

**Table 6:** Cell surface hydrophobicity (CSH) of isolated LAB

Isolate no.	Isolate identification	CSH (%)	Isolate no.	Isolate identification	CSH (%)
PRS^1^	*Lb. acidophilus* ATCC 20552	26.44± 2.31	L4	*Lb. fermentum*	36.15± 6.0
L1	*E. mediterraneensis*	23.24± 6.63	L5		38.88± 8.07*
L2		15.44± 6.72	L8		25.40± 2.4
L3		37.87± 9.71*	L9		20.87± 4.93
L11		23.46± 3.19	L10		24.29± 1.53
L12		24.22± 2.33	L13	*Strep. lutetiensis*	39.79+ 2.87*
L15		23.04± 6.22	L14		25.95± 3.01
L16		19.33± 2.93			

1: probiotic reference strain. Data are expressed as mean ± SD (standard deviation). * *P* values were ˂ 0.05.

#### Auto-aggregation assay

Generally, the results confirmed increasing the capability of all LAB isolates to aggregate with extending the incubation time (Table 7). Within the same time, there are significant differences between some isolates for their auto-aggregation capability. The strongest auto-aggregation ability, higher than 40% after 24 h, was observed for all *E. mediterraneensis* isolates except L3, and all *Lb. fermentum* isolates except L4. The highest auto-aggregation percentages of 64.71 ± 0.95, 63.61 ± 2.65, and 61.74 ± 1.8% were recorded for *E. mediterraneensis* (L11)*, Lb. fermentum* (L10), and *Strep. lutetiensis* (L14), respectively.

**Table 7:** Auto-aggregation capability of potential probiotic LAB

Isolate no.	Isolate identification	Auto-aggregation (%)			
		1 h	2 h	3 h	24 h
PRS^1^	*Lb. acidophilus* ATCC 20552	5.12 ± 0.89****	13.83 ± 1.43****	44.09 ± 2.22****	60.49 ± 2.63****
L1	*E. mediterraneensis*	3.82 ± 0.11***	15.24 ± 3.51***	27.96 ± 3.89***	50.25 ± 3.33***
L2		6.08 ± 3.36****	16.64 ± 2.62****	33.29 ± 2.78****	62.76 ± 1.13****
L3		4.66 ± 1.58**	9.33 ± 3.17**	21.46 ± 3.42**	28.92 ± 2.12**
L11		5.88 ± 0.08****	14.69 ± 1.36****	51.47 ± 0.75****	64.71 ± 0.95****
L12		5.11 ± 0.81****	12.27 ± 2.89****	41.51 ± 2.15****	60.5 ± 1.08****
L15		4.63 ± 0.11****	13.34 ± 3.59****	40.65 ± 3.79****	63.91 ± 2.06****
L16		5.84 ± 1.81****	16.49 ± 1.92****	40.93 ± 3.32****	60.11 ± 1.29****
L4	*Lb. fermentum*	6.0 ± 0.10**	13.01 ± 1.86**	23.02 ± 2.11**	34.99 ± 1.53**
L5		6.54 ± 1.83**	12.13 ± 2.14**	22.07 ± 2.6**	41.46 ± 3.07**
L8		4.18 ± 0.93***	15.20 ± 2.62***	35.59 ± 1.27***	51.26 ± 2.53***
L9		6.54 ± 1.59***	16.35 ± 3.01***	40.98 ± 1.42***	55.19 ± 2.08***
L10		4.61 ± 0.06****	15.38 ± 0.23****	51.79 ± 0.44****	63.61 ± 2.65****
L13	*Strep. lutetiensis*	6.75 ± 1.36**	12.58 ± 1.13**	20.21 ± 6.76**	35.78 ± 5.11**
L14		5.87 ± 1.41****	11.76 ± 0.17****	39.68 ± 2.04****	61.74 ± 1.8****

1: probiotic reference strain. Data are expressed as mean ± SD (standard deviation). * *P* values were ˂ 0.05.

#### Co-aggregation assay

The co-aggregation ability of LAB with bacterial pathogens *Sal. enterica* subsp. *enterica* serovar Typhimurium ATCC 14028, *E. coli* O157:H7 BCR 594, *S. aureus* subsp. *aureus* ATCC 25923 and *B. cereus* BCR 528 were evaluated (Tables 8 and 9). Similar to auto-aggregation, the co-aggregation ability with all selected bacterial pathogens was directly proportional with the incubation time. Also, it was observed that there are significant differences between some isolates for their co-aggregation capability during the same incubation time.

Obviously, the results of Table 8 indicated that the co-aggregation capability of *E. mediterraneensis* (L12), *Lb. fermentum* (L10), and *Strep. lutetiensis* (L13 and L14) with *Sal. typhimurium* has not changed for 20 h. After 24 h, the co-aggregation ability of *E. mediterraneensis* and *Lb. fermentum* with *Sal. typhimurium* ranged from 16.39 ± 7.91 to 62.95 ± 1.35% and from 20.04 ± 3.72 to 49.45 ± 1.25%, respectively. The strongest co-aggregation of 62.95 ± 1.35, 49.45 ± 1.25, and 45.16 ± 0.63% were recorded for *E. mediterraneensis* (L2), *Lb. fermentum* (L8), and *Strep. lutetiensis* (L14), correspondingly.

**Table 8:** Co-aggregation ability between isolated LAB and enteric bacteria *Sal. typhimurium* and *E. coli* O157:H7

Isolate no.	Isolate identification	Co-aggregation (%) with ***Sal. typhimurium***			Co-aggregation (%) with ***E. coli*** O157:H7		
		2 h	4 h	24 h	2 h	4 h	24 h
PRS^1^	*Lb. acidophilus* ATCC 20552	43.43 ± 0.38****	53.74 ± 2.25****	56.02 ± 1.62****	41.65 ± 3.21****	46.81 ± 2.79****	52.51±3.62****
L1	*E. mediterraneensis*	30.16 ± 2.75**	38.35 ± 2.74**	50.31 ± 2.72**	18.86 ± 0.36*	22.63 ± 1.72*	27.02 ± 1.77*
L2		34.75 ± 3.42***	40.27 ± 2.91***	62.95 ± 1.35***	31.48 ± 1.13**	36.99 ± 1.48**	39.73 + 2.02**
L3		10.29 ± 5.98*	15.57 ± 6.71*	16.39 ± 7.91*	11.19 ± 5.68*	19.28 ± 4.33*	25.29 ± 7.43*
L11		29.64 ± 4.26**	42.41 ± 0.66**	53.1 ± 3.18**	12.61 ± 1.67*	20.12 ± 4.94*	27.78 ± 2.96*
L12		50.51 ± 2.59****	55.69 ± 2.42****	55.69 ± 2.42****	39.03 ± 2.28****	44.82 ± 0.18****	51.66 ± 2.88****
L15		39.47 ± 1.2****	48.1 ± 1.23****	62.13 ± 2.73****	41.58 ± 2.47****	48.10 ± 1.23****	53.53 ± 1.33****
L16		44.92 ± 2.93****	47.33 ± 2.36****	55.60 ± 1.78****	20.66 ± 3.74*	27.17 ± 3.08*	32.49 ± 2.96*
L4	*Lb. fermentum*	7.99 ± 6.32*	17.04 ± 3.67*	20.04 ± 3.72*	9.89 ± 6.15*	15.85 ± 5.68*	22.95 ± 4.26*
L5		9.45 ± 6.79*	20.48 ± 1.79*	26.53 ± 3.03*	5.85 ± 3.46*	13.56 ± 3.07*	20.29 ± 5.34*
L8		37.99 ± 3.14***	47.81 ± 1.27***	49.45 ± 1.25***	31.48 ± 2.74**	33.11 ± 2.7**	35.85 ± 0.77**
L9		34.30 ± 2.83***	39.78 ± 4.46***	42.84 ± 5.24***	18.4 ± 1.76**	29.98 ± 3.76**	35.51 ± 3.61**
L10		43.54 ± 1.4****	48.38 ± 1.4****	48.38 ± 1.4****	23.1 ± 1.56*	24.71 ± 1.54*	27.94 ± 1.5*
L13	*Strep. lutetiensis*	9.03 ± 6.01*	18.09 ± 4.85*	18.09 ± 4.85*	11.11 ± 3.5*	16.17 ± 1.79*	24.22 ± 2.76*
L14		40.32 ± 2.84***	45.16 ± 0.63***	45.16 ± 0.63***	31.92 ± 0.69***	38.53 ± 2.01***	45.76 ± 1.92***

1: probiotic reference strain. Data are expressed as mean ± SD (standard deviation). * *P* values were ˂ 0.05.

For *E. coli*, the co-aggregation ability ranged from 25.29 ± 7.43 to 53.53 ± 1.33% and from 20.29 ± 5.34 to 35.85 ± 0.77% for *E. mediterraneensis* and *Lb. fermentum*, respectively. The highest co-aggregation, represented by 53.53 ± 1.33, 35.85 ± 0.77, and 45.76 ± 1.92%, were demonstrated for *E. mediterraneensis* (L15), *Lb. fermentum* (L8), and *Strep. lutetiensis* (L14), respectively.

For toxigenic bacteria, *S. aureus* and *B*. *cereus*, the results indicated that the maximum co-aggregation ability of *E. mediterraneensis* (L12) with *S. aureus* reached after 4 h and continued until 24 h (Table 9). After 24 h, the co-aggregation ability of *E. mediterraneensis* with *S*. *aureus* and *B*. *cereus* ranged from 15.62 ± 5.57 to 44.29 ± 2.96% and from 16.39 ± 7.91 to 49.68 ± 2.65%, respectively. On the other hand, *Lb. fermentum* isolates had the ability to aggregate with *S*. *aureus* and *B*. *cereus* in a range of 10.09 ± 3.62 to 45.12 ± 3.56% and 24.86 ± 5.33 to 51.58 ± 2.39%, correspondingly. The greatest co-aggregation of 44.29 ± 2.96, 45.12 ± 3.56, and 37.32 ± 1.14% was observed between *S. aureus* and each of *E. mediterraneensis* (L15), *Lb. fermentum* (L10), and *Strep. lutetiensis* (L14), individually. For *B. cereus*, the strongest co-aggregation, signified by 49.68 ± 2.65, 51.58 ± 2.39, and 40.31 ± 2.84%, were established with *E. mediterraneensis* (L15), *Lb. fermentum* (L10), and *Strep. lutetiensis* (L14), respectively.

**Table 9:** Co-aggregation ability between isolated LAB and toxigenic bacteria *S. aureus* and *B. cereus*

Isolate no.	Isolate identification	Co-aggregation (%) with ***S. aureus***			Co-aggregation (%) with ***B. cereus***		
		2 h	4 h	24 h	2 h	4 h	24 h
PRS	*Lb. acidophilus* ATCC 20552	38.8 ± 4.42***	40.52 ± 4.39***	43.38 ± 3.52***	38.8 ± 4.42***	41.07 ± 5.26***	44.5 ± 5.20***
L1	*E. mediterraneensis*	32.69 ± 0.63***	34.58 ± 0.61***	38.98 ± 0.58***	18.24 ±1.14*	22.63 ± 1.72*	27.02 ± 1.77*
L2		38.61 ± 2.97****	39.69 ± 3.67****	44.13 ± 2.7****	24.76 ± 4.17**	30.87 ± 2.86**	34.19 ± 2.75**
L3		9.48 ± 4.61*	12.15 ± 4.4*	15.62 ± 5.57*	11.10 ± 7.37*	16.39 ± 7.91*	16.39 ± 7.91*
L11		29.04 ± 3.87**	30.94 ± 3.82**	34.09 ± 3.76**	29.62 ± 4.57***	34.76 ± 1.52***	47.39 ± 3.34***
L12		39.58 ± 3.35***	41.31 ± 3.3***	41.31 ± 3.3***	40.77 ± 1.29***	42.5 ± 1.24***	47.63 ± 2.73***
L15		41.05 ± 3.00****	42.67 ± 2.98****	44.29 ± 2.96****	41.56 ± 3.07****	44.82 ± 2.76****	49.68 ± 2.65****
L16		29.5 ± 4.58**	32.48 ± 3.57**	34.85 ± 3.58**	39.00 ± 2.51***	43.18 ± 1.41***	48.46 ± 3.14***
L4	*Lb. fermentum*	10.85 ± 6.7*	14.89 ± 4.81*	17.90 ± 4.71*	5.91 ± 2.72*	15.85 ± 5.68*	24.86 ± 5.33*
L5		5.85 ± 3.46*	7.52 ± 3.79*	10.09 ± 3.62*	7.43 ± 6.08*	20.48 ± 1.79*	26.53 ± 3.03*
L8		39.09 ± 1.89****	40.73 ± 1.86****	44.55 ± 1.74****	34.76 ± 1.87***	39.65 ± 1.80***	48.91 ± 1.25***
L9		34.93 ± 2.24***	36.76 ± 2.2***	38.61 ± 2.15***	35.55 ± 2.09***	40.45 ± 2.11***	45.97 ± 1.97***
L10		40.29 ± 2.27****	41.91 ± 2.25****	45.12 ± 3.56****	33.31 ± 1.41***	44.58 ± 3.35***	51.58 ± 2.39***
L13	*Strep. lutetiensis*	11.11 ± 3.5*	15.13 ± 2.86*	20.15 ± 4.32*	14.05 ± 4.24*	17.08 ± 4.14*	21.12 ± 4.78*
L14		33.68 ± 2.8**	35.48 ± 2.75**	37.32 ± 1.14**	34.89 ± 2.96**	37.28 ± 3.59**	40.31 ± 2.84**

1: probiotic reference strain. Data are expressed as mean ± SD (standard deviation). * *P* values were ˂ 0.05.

#### Antimicrobial activity assay

Three extracts, prepared from each isolated LAB, were employed to assess the antibacterial activity against aforementioned pathogenic bacteria. Extract 1 was crude extract that might contain H_2_O_2_, organic acids, and bacteriocins; extract 2 was crude extract that could contain organic acids and bacteriocins, whereas extract 3 was crude bacteriocin. The results in Table 10 revealed that all extracts prepared from *E. mediterraneensis* (L11, L15, and L16) and *Lb. fermentum* (L9) displayed antagonistic activity against all selected bacterial pathogens with inhibition zone which ranged from 11 to 29 mm in diameter. Also, all extracts of *E. mediterraneensis* (L12), *Lb*. *fermentum* (L8), and *Strep*. *luteliensis* (L14) exhibited antibacterial effect against all pathogens, except *Sal. typhimurium*, *E*. *coli* O157:H7, and *B. cereus*, respectively. All extracts of *Lb. fermentum* (L10) had antimicrobial effect against *Sal. typhimurium* and *S. aureus* only.

**Table 10:** Inhibitory effect of the extracts prepared from isolated LAB

Isolate no.	Isolate identification	Diameter of inhibition zone (mm)											
		***Sal. typhimurium***			***E. coli*** O157:H7			***B. cereus***			***S. aureus***		
		E1	E2	E3	E1	E2	E3	E1	E2	E3	E1	E2	E3
PRS^1^	*Lb. acidophilus* ATCC 20552	23	21	11	24	22	11	21	19	12	22	21	11
L1	*E. mediterraneensis*	16	15	R	12	12	R	13	12	R	12	11	R
L2		14	12	R	13	11	R	14	14	R	12	11	R
L3		R	R	R	R	R	R	R	R	R	R	R	R
L11		23	21	13	19	17	13	20	19	12	19	17	13
L12		15	13	R	22	22	12	15	14	11	16	15	11
L15		25	24	14	29	28	12	18	13	11	19	17	11
L16		24	24	12	17	16	13	19	20	11	18	16	12
L4	*Lb. fermentum*	R	R	R	R	R	R	R	R	R	R	R	R
L5		R	R	R	R	R	R	R	R	R	R	R	R
L8		18	19	12	R	R	R	22	20	15	19	17	11
L9		20	21	13	17	16	12	15	14	11	16	15	11
L10		16	15	11	R	R	R	R	R	R	18	16	12
L13	*Strep. luteliensis*	R	R	R	R	R	R	R	R	R	R	R	R
L14		22	21	12	21	19	11	14	12	R	19	17	11

All indicator pathogenic bacteria were entirely resistant to *E. mediterraneensis* (L3), *Lb. fermentum* (L4 and L5) and *Strep. lutetiensis* (L13). Extract 3 prepared from *E. mediterraneensis* (L1 and L2) had no antagonistic activity against all tested pathogens.

## Discussion

Generally, lactic acid bacteria (LAB) are the main group of probiotics used for humans and animals. In the food fermentation, they play a significant role by inhibiting the growth of spoilage/pathogenic microorganisms, and by producing fermented food products with desired flavor, aroma, and texture.

Usually, the probiotic microorganisms are screened from food and nonfood sources. The nonfood sources include gastrointestinal tract, as the main nonfood source, honeycomb, soil and plant surface. On the other hand, the food sources are represented by fermented dairy, meat and vegetable products, and fruit juices.

In the present study, after performing Gram staining, catalase test and blood hemolysis test, the DNA fingerprinting and 16S rRNA gene sequencing were applied to identify 16 acid and bile salt-tolerant bacteria isolated from different food sources. The results revealed that, 43.75% were identified as *Enterococcus mediterraneensis* isolated from rayeb milk, kareish cheese, and frankfurte. Also, 31.25% were classified as *Lactobacillus fermentum* isolated from rayeb milk, yoghurt, and frankfurter. Both *Streptococcus lutetiensis* and *Bacillus circulans* represented 12.5% of the total isolates. *Enterococcus mediterraneensis* was first isolated and identified in 2019 from the stool of a 39-year-old male Pygmy in the Democratic Republic of Congo [21]. There are no previous studies reporting isolation of *E. mediterraneensis* from Egyptian sources. Therefore, this study is considered as the first one to isolate *Enterococcus mediterraneensis* in Egypt and to characterize its probiotic properties worldwide.

*Streptococcus lutetiensis* is belonging to *Streptococcus bovis/Streptococcus equinus* complex (SBSEC) which is a non-enterococcal group D *Streptococcus* spp. complex. The strains of SBSEC are commensal colonizers of the gastrointestinal tract of humans and animals including ruminants as cattle, sheep, goats, and camels. Some strains of SBSEC have been associated with different diseases as endocarditis, bacteremia, biliary tract, prosthetic joint infections, meningitis, and diarrhea. Additionally, some strains are considered as important species having a main role in the quality of fermented food products. Moreover, some SBSEC strains as *Strep. lutetiensis* and *Strep. gallolyticus* subsp. *macedonicus* are consumed as a part of the daily diet. Consequently, they are considered to be safe for human consumption [22, 23]. For blood hemolysis activity, some strains are gamma-hemolytic (non-hemolytic), which agreed with the results of the present study, and some exhibit alpha-hemolytic activity.

The representatives of various *Bacillus* species have a long history of safe use as probiotics. Globally, there is a variety of commercial formulations containing *Bacillus* spp. to be used as probiotics [24]. *Bacillus circulans*, reported to cause human infection, is a member of the *Bacillus subtilis* group [25]. Most species of this group exhibit β-hemolytic activity. In this study, *Bacillus circulans* was isolated from frankfurter and exhibited γ-hemolysis on blood agar. This result was in agreement with the findings of Alebouyeh et al. [26] who isolated nonhemolytic *B. circulans* from a 62-year-old patient with 4 years of unknown end-stage renal disease. It is known that *Bacillus circulans* is an opportunistic pathogen found in soil, sewage, and food. Also, many previous studies isolated *B. circulans* from cases of meningitis, prosthetic heart valve [27], endocarditis, endophthalmitis [28], and wound infection. There are other reports indicating that *B. circulans* is a causative agent of sepsis in immunocompromised hospitalized patients [24].

This study reported the isolation of *Clavispora lusitaniae* from kareish cheese. *Clavispora lusitaniae*, which is also known as *Candida lusitaniae*, could be isolated from different sources as digestive tract, fruit juices, citrus peel, and milk from cow infected with mastitis. Generally, *Clavispora lusitaniae* is considered as a nosocomial pathogen [29].

Generally, the microorganisms are considered as safe and beneficial probiotics for human and animal use after their proper identification and characterization. Consequently, *in vitro* characterization of safety and functional properties is extremely imperative for the selection of highly effective probiotic strains. In the current study, the functional assays to evaluate the probiotic efficiency of isolated *B. circulans* and *Clavispora lusitaniae* were not performed because they are stated as microbial pathogens as was previously mentioned.

Lacking the hemolytic activity is one of the most important safety characteristics recommended by FAO/WHO (2002) for probiotic microorganisms to be considered as food grade bacteria [30]. Actually, all lactic acid bacterial isolates (14 isolates) were nonhemolytic isolates. The absence of hemolytic activity of isolated *E. mediterraneensis* was in agreement with the previous study of Takakura et al. [21].

Evaluating the antibiotic susceptibility is considered as the second important safety aspect regarding employing the bacteria as probiotics in food and animal feed. The results revealed that the isolates of *E. mediterraneensis* were susceptible to ampicillin and ceftolozane inhibiting the cell wall biosynthesis, and to neomycin inhibiting the protein synthesis, unlike the other enterococci reported by Miller et al. [31] to have innate resistance to antibiotics of β-lactams and aminoglycosides. All *Lb. fermentum* isolates exhibited susceptibility to ceftolozane, neomycin, and sulphamethoxazole inhibiting the cell wall and protein biosynthesis and folic acid metabolism, respectively. These results were in agreement with previous studies of Danielsen and Wind [32] and Abriouel et al. [33]. The two isolates of *Strep. lutetiensis* displayed sensitivity to antibiotics with mode of action to inhibit the biosynthesis of cell wall and proteins.

Antibiotic resistance of probiotics and absence of transferable antibiotic resistance genes, that could be transferred horizontally to other bacteria, are imperative to avoid the risk of prevalence of antibiotic resistance genes in the environment and to confirm the safety of probiotic application as food and feed additives [34]. European Centre for Disease Prevention and Control (ECDC) and the Centers for Disease Control and Prevention (CDC) defined the multidrug resistance (MDR) as the resistance to at least one agent in three or more antimicrobial categories [35]. According to this definition, *E*. *mediterraneensis* isolates (L3, L11, L12, and L16), all isolated *Lb. fermentum* and *Strep. lutetiensis* are regarded as multidrug-resistant bacteria. Generally, lactobacilli are known to have intrinsic resistance to vancomycin [36, 37]. The genes encoding its resistance are located on the chromosome which indicates these genes are not transferred horizontally. On the other hand, genes encoding the tetracycline resistance are often located on the conjugative plasmids [38]. Therefore, they could be transferred.

Phenol and its derivatives are known to have antibacterial and antifungal activity. Therefore, evaluating the resistance of potential probiotics to phenol is significant to be applied in animal and fish feeding as these compounds are produced in their intestine by bacterial deamination of aromatic amino acids liberated during digestion of dietary proteins [39]. In the present study, the highest survival rate to 0.4% phenol was recorded with *E. mediterraneensis* (L16), *Lb. fermentum* (L9), and *Strep. lutetiensis* (L14).

Survival in the gastrointestinal juice, cell surface hydrophobicity (CSH), and capability to auto-aggregate are the foremost selective traits of potential probiotics to be functionally effective in the host. Evaluating the performance of probiotic candidates in simulated gastrointestinal environment is essential to sufficiently predict their *in vivo* behavior as without this property the microorganisms will not be functionally influential [17]. Some studies have evaluated the resistance of probiotics to the gastrointestinal juices through using the gastric and intestinal juices individually [16, 40]. In this study, the successive gastric and intestinal digestion was employed to simulate the physiological conditions of human and animal gastrointestinal digestion. The simulated gastric conditions were characterized by the presence of 0.3% bile salt and 0.1% pepsin enzyme in acidic conditions (pH 2.5), whereas the simulated intestinal conditions were represented by the presence of 1% pancreatin containing trypsin, lipase, protease, and amylase enzymes in higher pH of 6.8. The tolerance of potential probiotics, used in fish aquaculture and animal feeding, to bile salt is substantial not only to confirm their ability to survive in the indigenous bile salt present naturally in fish and animal intestine, but also to that added to animal and fish feeds. Recently, the plant feed ingredients, supplemented with bile salt, are employed to replace fishmeal and fish oil in feed production. This supplementation is very essential because some compounds for bile salt synthesis, as cholesterol and taurine, are usually insufficient in plant feed ingredients [41]. The results revealed that some isolates have high survivability that reached 23.9 ± 1.85 and 32.73 ± 0.84% for *Lb. fermentum* (L8) and *E. mediterraneensis* (L2), respectively. Conversely, the low survival rates of 2.01 ± 0.01 and 1.35 ± 0.06% were recorded for *Lb. fermentum* (L9) and *E. mediterraneensis* (L16), correspondingly. The low survivability may be attributed to the antimicrobial effect of bile salts which causes permeabilization of the bacterial cell membrane and leakage of cytosol consequently. Some researchers reported that the effect of bile salt on the bacterial cytoplasmic membrane depends on its concentration. The high concentrations dissolve membrane lipids, causing leakage of cell materials and cell death. Low concentrations have less undesirable effects on the membrane fluidity and permeability by changing membrane proteins or increasing transmembrane divalent cation flow [42]. According to Botta et al. [17] who reported that the microorganisms with ODS less than 0.00001%, after sequential transfer from gastric to intestinal juice, are not considered resistant to the gastrointestinal conditions, all *E. mediterraneensis, Lb. fermentum,* and *Strep. lutetiensis* isolates have an extremely considerable resistance as the lowest ODS value was 1.35 ± 0.06%.

The high cell surface hydrophobicity and strong auto-aggregation capability are considered as essential requirements of probiotics to ensure strong colonization and adhesion to intestinal epithelium of the host to provide their health benefits. Also, the strong adhesion to mucosal surfaces and epithelial cells of the gastrointestinal tract allows probiotics to overcome the gastric motility and therefore enhances the interactions between probiotic bacteria and host [43]. Generally, the cell surface hydrophobicity is different between bacterial species, but there are numerous compounds playing a main role in the bacterial CSH. These compounds include lipoteichoic acid, core oligosaccharides, outer membrane proteins and lipids, surface fibrils and several fimbriae [43]. Hydrophobicity is likely due to a complex interaction between positively charged, negatively charged, hydrophilic and hydrophobic components on the bacterial surface [44]. The studies of Jena et al. [16] and Abdulla et al. [44] reported that the bacterial strains, with more than 40% hydrophobicity, will be considered hydrophobic. According to their findings, *E. mediterraneensis* (L3), *Lb*. *fermentum* (L5), and *Strep*. *lutetiensis* (L13) could be considered as hydrophobic isolates as their CSH, ranging from 37.87 ± 9.71 to 39.79 ± 2.87%, is slightly less than 40%.

Bacterial auto-aggregation is defined as the ability of bacteria of the same strain to bind to themselves. This phenomenon is observed clearly through the formation of bacterial clumps that precipitate at the tube bottom. Generally, the auto-aggregation is mediated by exopolysaccharide and surface proteins as extracellular serine/threonine-rich protein of *Lb. plantarum* NCIMB 8826 [45] and S-layer proteins of *Lb. acidophilus* M92 [46]. The current study confirmed the findings of other studies reported that the self-aggregation increases with extending the incubation time [16, 44]. The greatest auto-aggregation ability, higher than 50%, was recorded with 71.43% of isolated LAB. In another study, the strongest auto-aggregation ability reached 47.2 ± 2.4% [16].

Although some studies [44, 47, 48] reported the direct correlation between bacterial cell surface hydrophobicity and auto-aggregation capability, the results of this study do not support the hypothesis as relation of the auto-aggregation and CSH was not characterized.

The auto-aggregation and co-aggregation (aggregation between genetically different strains) are considered as key properties of probiotics to prevent colonization of gastrointestinal tract with pathogens. This is due to the formation of biofilms of auto-aggregating bacteria on the intestinal mucosa and intercellular adhesion between co-aggregating bacteria and microbial pathogens [49]. Thence, aggregation could be counted as one of the defense mechanisms of host for anti-infection. In this work, the *E. mediterraneensis* isolates (L2, L12, and L15) with high auto-aggregation ability exhibited high co-aggregation capability with *Sal*. *typhimurium*, *E*. *coil* O157:H7, *S*. *aureus,* and *B*. *cereus* in a range of 55.69 ± 2.42 to 62.95 ± 1.35, 39.73 ± 2.02 to 53.53 ± 1.33, 41.31 ± 3.3 to 44.29±2.96 and 34.19 ± 2.75 to 49.68 ± 2.65%, respectively. Also, the *Lb. fermentum* isolates (L8, L9, and L10) with high auto-aggregation ability exhibited high co-aggregation with *Sal*. *typhimurium*, *E*. *coil* O157:H7, *S*. *aureus,* and *B*. *cereus* in a range of 42.84 ± 5.24 to 49.45 ± 1.25, 27.94 ± 1.5 to 35.85 ± 0.77, 38.61 ± 2.15 to 45.12 ± 3.56 and 45.97 ± 1.97 to 48.91 ± 1.25%, individually. The isolate *Strep*. *lutetiensis* (L14) displayed high auto-aggregation of 61.74 ± 1.8% and co-aggregation of 45.16 ± 0.63, 45.76 ± 1.92, 37.32 ± 1.14, and 40.31 ± 2.84% with *Sal*. *typhimurium*, *E*. *coil* O157:H7, *S*. *aureus,* and *B*. *cereus,* correspondingly. Thus, these isolates possessing the strong ability to auto-aggregate and co-aggregate pathogens could be valuable to the intestinal health.

The relation between probiotics and pathogenic bacteria depends on the co-aggregation and antimicrobial activity of probiotics to inhibit pathogens. The isolates of prospective probiotic LAB should exhibit manifest antimicrobial activities against pathogenic bacteria causing diseases in human and animal gut to improve the host health and to balance the gut microbiota. In this study, 85.71 and 50% of *E*. *mediterraneensis* and *Strep. lutetiensis* isolates, respectively displayed antibacterial activity against all selected bacterial pathogens. For *Lb. fermentum* isolates, 40 and 20% had effect against *B. cereus* and *E. coli* O157: H7, correspondingly. Furthermore, 60% were active against both *Sal. typhimurium* and *S. aureus*. The antibacterial activity of extracts prepared from LAB was evaluated to specify the antimicrobial agents. No inhibitory effect was observed from extract 3 prepared from *E. mediterraneensis* (L1 and L2), (L12), and *Strep. luteliensis* (L14) against all tested pathogens, *Sal. typhimurium* and *B. cereus*, respectively. Absence of the antagonistic activity of the third extract indicates the antimicrobial effect attributed mainly to organic acids only or organic acids and H_2_O_2_ as organic acids are present in the first and second extracts but H_2_O_2_ is found in the first extract only. According to the obtained results, 57.14% of *E. mediterraneensis* isolates (L11, L12, L15, and L16) could be considered bacteriocinogenic against *E. coli* O157:H7, *B. cereus,* and *S. aureus*, whereas 42.86% (L11, L15, and L16) was bacteriocinogenic against *Sal. typhimurium*. For *Lb. fermentum*, 60% (L8, L9, and L10) could be characterized as bacteriocinogenic isolates against *Sal. typhimurium* and *S. aureus*, 40% (L8 and L9) against *B. cereus* and finally 20% (L9) against *E. coli* O157:H7. Also, *Strep. lutetiensis* (L14) could be specified as bacteriocinogenic against all tested bacterial pathogens. Many studies demonstrated the antagonistic effect of LAB against different pathogens through production of various antimicrobial agents including carbon dioxide, hydrogen peroxide, organic acids, and bacteriocins [17, 40, 43].

## Conclusion

The results collectively revealed that *E*. *mediterraneensis* (L2, L12, and L15), *Lb. fermentum* (L8, L9, and L10), and *Strep. lutetiensis* (L14) could be considered excellent probiotic candidates as they have promising properties essential for potential probiotics. *In vivo* experiments are recommended to evaluate functionality of the most promising isolates in human and animals. Actually, these experiments are considered as additionally safety tests to check all possibilities of using described probiotic species for human and animal diet supplementation.

## Abbreviations

*Lb*: *Lactobacillus*
*E*. *mediterraneensis*: *Enterococcus mediterraneensis*
*Strep. lutetiensis*: *Streptococcus lutetiensis*
AM: Ampicillin
CEF: Ceftolozane
CLI: Clindamycin
TE: Tetracycline
MXF: Moxifloxacin
NEO: Neomycin
SXT: Sulphamethoxazole
VAN: Vancomycin
CHL: Chloramphenicol
R: Resistant
S: Sensitive
I: Intermediate resistant
